# Extract from Proceedings of the Mississippi Valley Dental Association

**Published:** 1867-06

**Authors:** 


### Proceedings of Societies.

### EXTRACT FROM PROCEEDINGS OF THE MISSISSIPPI VALLEY DENTAL ASSOCIATION.

THOUGH the substantial and practical details of science and art should ever take the lead in meetings of the profession, yet members of the profession are men—hence they have *feelings* and affections, as well as reasoning faculties. When a meeting so results as to bless both the emotional and intellectual faculties of the members, it is a good meeting. Not many societies have been permitted to enjoy so pleasant an incident as that recorded below. Years ago a veteran member of the Association retired from practice, and, therefore, from active membership. Having resumed practice, of course he resumed his place in the Association, and having tried him before, the old society knows exactly what to do with him. We couldn't think of waiting on the tedious round of rotation, before putting him in his proper place. Many of us don't know how to preside, and we know it; he does know how, and we know that too. And to see him in the chair as fresh and buoyant—yes, even as *boy*-ant as when there before, made us all feel younger, and more ready to gird on the armor of professional science, to battle in the cause of truth and progress.

Still gently deal with him, O time. W.

### VALEDICTORY BY PRESIDENT G. W. KEELY.

DR. KEELY, on leaving the chair, said: "In retiring from this office to which I was elected by you, it only remains for me to thank you for your kind consideration, and the assistance you have rendered me. I now have the pleasure to introduce to you, one who is an old wheel-horse in the profession, who retired from the profession several years ago, and whom we all heartily welcome back."

The retiring President then introduced Dr. W. H. GODDARD, who was received with hearty applause. On taking the chair Dr. Goddard said:

"Gentlemen of the Mississippi Valley Dental Association: this honor was not only unexpected by me, but had I known your intention to confer it, I should have absented myself from here this afternoon. I had made arrangements to go away at 4 o'clock, and had my baggage started on the way, but being here and learning your wishes, I am, as I have always endeavored to be, ready to do that which my professional brethren desire. No matter how hard or severe the work, or how incompetent I may be, I know I will have your assistance. I thank you, brethren, for the honor conferred. I have had this office once before. There are those here who will remember twelve or fourteen years ago, I was called to this high position. I endeavored to fill it to the best of my ability then, and will endeavor to do so now.

Having filled the office once, I thought there was no danger of being called on again, till you had gone all through the ranks; for I think there are those here who are much more competent to fill this chair—not fill the *chair*—I can do that, I have corpulency enough for that—but that can perform the duties pertaining to this office better than I can. I hope, however, to receive your kind indulgence, your advice and assistance."

[The Doctor on taking his seat was again greeted with hearty applause.]

The subject of receiving members being under consideration.

Dr. Berry said, I do not take the ground, that a candidate for membership here, or in any other society, ought to be rejected because he is not good looking. But I do insist that candidates for membership should undergo a thorough examination. With one of the founders of this Association, I think we have been too lax generally in this matter. I think the time has come when we ought to be more rigid.

It will not do to admit members here with the qualifications we did twenty years ago.

The standard must be elevated and advanced as the profession advances. We must require a fair degree of excellence in the qualifications of a candidate, who now applies for membership.

I may be censured for some of my ideas on this subject, but I can stand it.

